# Improved productivity of poly (4-hydroxybutyrate) (P4HB) in recombinant *Escherichia coli* using glycerol as the growth substrate with fed-batch culture

**DOI:** 10.1186/s12934-014-0131-2

**Published:** 2014-08-31

**Authors:** Sylvaine Le Meur, Manfred Zinn, Thomas Egli, Linda Thöny-Meyer, Qun Ren

**Affiliations:** Laboratory for Bioactive Materials, Swiss Federal Laboratories for Materials Science and Technology (Empa), Lerchenfeldstrasse 5, St. Gallen, CH-9014 Switzerland; Swiss Federal Institute of Technology Zurich (ETH), Environmental Sciences, Rämistrasse 101, Zurich, 8092 Switzerland; Biotechnology, HES-SO Valais Wallis, Rue du Rawyl 64, P.O.B. 2134, Sion, CH-1950 Switzerland; Environmental Microbiology, Swiss Federal Institute of Aquatic Science and Technology (Eawag), Überlandstrasse 133, P.O. Box 610, Dübendorf, CH-8600 Switzerland

**Keywords:** P4HB, High cell density culture, Glycerol, Acetic acid, Recombinant *E. coli*, Fed batch, Productivity

## Abstract

**Background:**

The most successful polyhydroxyalkanoate (PHA) in medical applications is poly(4-hydroxybutyrate) (P4HB), which is due to its biodegradability, biocompatibility and mechanical properties. One of the major obstacles for wider applications of P4HB is the cost of production and purification. It is highly desired to obtain P4HB in large scale at a competitive cost.

**Results:**

In this work, we studied the possibility to increase P4HB productivity by using high cell density culture. To do so, we investigated for the first time some of the most relevant factors influencing P4HB biosynthesis in recombinant *Escherichia coli*. We observed that P4HB biosynthesis correlated more with limitations of amino acids and less with nitrogen depletion, contrary to the synthesis of many other types of PHAs. Furthermore, it was found that using glycerol as the primary carbon source, addition of acetic acid at the beginning of a batch culture stimulated P4HB accumulation in *E. coli*. Fed-batch high cell density cultures were performed to reach high P4HB productivity using glycerol as the sole carbon source for cell growth and 4HB as the precursor for P4HB synthesis. A P4HB yield of 15 g L^−1^ was obtained using an exponential feeding mode, leading to a productivity of 0.207 g L^−1^ h^−1^, which is the highest productivity for P4HB reported so far.

**Conclusions:**

We demonstrated that the NZ-amines (amino acids source) in excess abolished P4HB accumulation, suggesting that limitation in certain amino acid pools promotes P4HB synthesis. Furthermore, the enhanced P4HB yield could be achieved by both the effective growth of *E. coli* JM109 (pKSSE5.3) on glycerol and the stimulated P4HB synthesis *via* exogenous addition of acetic acid. We have developed fermentation strategies for P4HB production by using glycerol, leading to a productivity of 0.207 g L^−1^ h^−1^ P4HB. This high P4HB productivity will decrease the total production cost, allowing further development of P4HB applications.

## Background

Polyhydroxyalkanoates (PHAs) are natural polyesters that have gained special interest due to their biodegradability and biocompatibility [1–4]. PHAs can be stored by a wide variety of microorganisms as intracellular reserve materials. They are accumulated when the bacterial cells experience nutrient-limited growth conditions other than carbon. Up to now, more than a hundred different monomers have been reported to be incorporated as building blocks into bacterial PHAs, resulting in different material properties of the polymers [5–8].

One of the most promising PHAs for medical applications is poly(4-hydroxybutyrate) (P4HB) [9]. This homopolymer is a strong and flexible material, which can be employed for instance for tissue engineering and drug delivery. In addition, P4HB is biocompatible and extremely well tolerated *in vivo* due to the fact that hydrolysis of P4HB yields 4HB, which is a common metabolite in the human body [10]. This biopolymer was the first − and so far only − PHA-based material approved for clinical application as absorbable suture (TephaFLEX®) by the FDA. Other applications of P4HB are currently under investigation, for example, Opitz and coworkers successfully produced an ovine, aortic blood vessel substitute using bioabsorbable P4HB scaffolds [11]. However, the high cost of P4HB hinders its wider applications [2]. In order to have sufficient material available for application studies and to reduce production cost, much research has been focused on the efficient production of P4HB by increasing the amount of biopolymer accumulated in the cells. Surprisingly, there are no reports found in the literature documenting the use of high cell density (higher than 20 g L^−1^) processes to reach high P4HB productivities. High productivity can be obtained by combining cultivation procedures to achieve maximum polymer accumulation per cell with those allowing fast growth to reach high cell densities. High cell density processes allow increasing the productivity of accumulated metabolites with simultaneously decreasing the production cost as a result of a lower culture volume (smaller bioreactors) and shorter fermentation time. So far there is no generally accepted value to be defined as high cell density [12]. Different studies have considered different values of cell dry weight (CDW), for example, Restaino and coworkers reported a high cell density of 22 g CDW per liter for *Escherichia coli* culture [13], whereas Yamanè and Shimizu mentioned that high cell density cultivation is achieved when reaching about cell concentrations of 50 g CDW per liter [14].

Generally, high cell densities are reached by fed-batch cultures using a pulse, linear or exponential feed of the limiting carbon substrate. It was reported that exponential feeding allows to achieve cell concentrations up to 148 g L^−1^ using glycerol as carbon source with *E coli* TG1 cells [15]. To increase productivity, it is important to understand the factors stimulating P4HB accumulation. In earlier work using recombinant *E. coli*, we identified three physiological phases during P4HB production: i) the “growth phase”, in which cells grew exponentially, ii) the “accumulation phase”, in which cells stopped dividing and started to accumulate P4HB, and iii) the “stagnation phase”, in which both cell proliferation and P4HB accumulation stopped while the total biomass remained constant [16]. Hence, under this condition P4HB synthesis was found to be distinctly separated from cell growth and to occur after exponential cell growth stopped. This is different from the synthesis of other types of PHAs in recombinant *E. coli* [3,17].

While the development of a highly efficient fermentation process constitutes one part of the optimization procedure, the use of a cheap carbon substrate is another crucial factor that allows reducing production costs significantly. For example, the hemicellulose derivative xylose can be used as an industrially relevant carbon source for growth of *E. coli* strains in general [18] and for P4HB homopolymer production in particular [16]. Glycerol is another interesting carbon source because it currently accumulates as a waste byproduct during biodiesel production [19], and therefore, production of higher value products from crude glycerol is of primary interest. Glycerol, which can be used both as carbon and energy source, enables cheap production of valuable synthons, for example 1,3-propanediol, dihydroxyacetone, ethanol, succinate, and propionate [20] and has been tested as growth substrate for *E. coli* in fed-batch processes to reach high cell density [21]. Advancements in metabolic engineering made it possible to produce many heterologous products such as proteins [22], biofuels [23], and PHAs [2,3] in *E. coli* strains at high cell density. A recent study demonstrated that crude and refined glycerol from biodiesel industry can be used as carbon substrate to accumulate medium-chain-length PHAs by *Pseudomonas mediterranea* and *P. corrugate* [24].

In this study we investigated the influence of different nutrient concentrations on P4HB synthesis in *E. coli* JM109 (pKSSE5.3), a strain harboring the genes essential for P4HB production from 4HB. We further tested whether or not refined glycerol can be used as the growth substrate for P4HB production in high cell density cultures. It was found that acetate can stimulate P4HB synthesis in recombinant *E. coli* grown on glycerol. Based on this study, an efficient process was developed to reach high productivity of P4HB by using high cell density cultures combined with acetic acid addition.

## Results and discussion

Previously, we observed that the recombinant *E. coli* strain JM109 (pKSSE5.3) synthesized only small amount of P4HB (about 10%) when glycerol was offered as carbon source [16]. In this study, we attempted to utilize this inexpensive carbon source as the growth substrate for P4HB synthesis by high cell density cultivation. To enhance P4HB production, we first set out to identify the influencing factors for P4HB synthesis. It is difficult to conclude whether a factor plays a significantly influencing role or not when the base value is low such as 10%, especially when the factor has negative impact. Thus, xylose, which could lead to 30-70% P4HB [16], was used as the growth carbon source for the investigation.

### Identification of factors influencing P4HB synthesis

**Figure 1:**
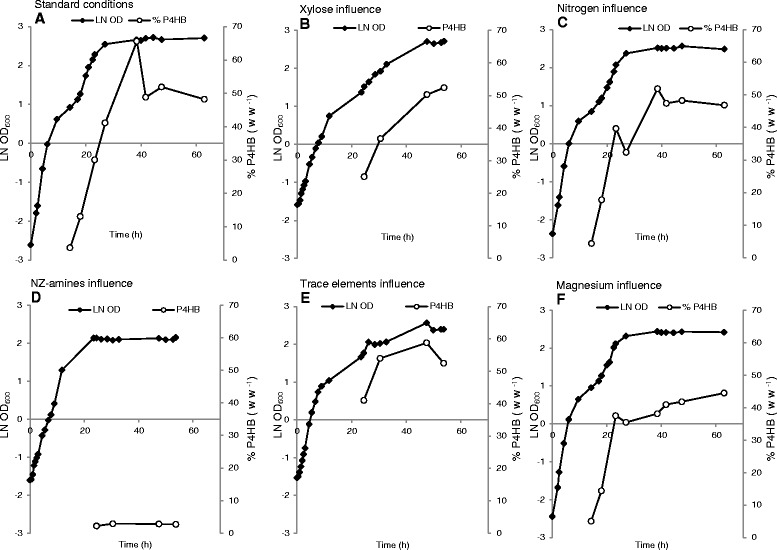
**Influence of various factors on growth and P4HB accumulation.** *E. coli* JM109 (pKSSE5.3) was grown in 1 L bioreactors in modified E2 medium. The modified E2 medium containing 10 g L^−1^ xylose, 4 g L^−1^ Na-4HB, 1 g L^−1^ NZ-amines, and 1 mL L^−1^ trace elements was used as standard medium. Culture **A**: standard medium; Culture **B**: two times more xylose was added to the standard medium, leading to 20 g L^−1^ xylose; Culture **C**: five times more nitrogen source NaNH_4_HPO_4_ · 4H_2_O was added to the standard medium, leading to a final NaNH_4_HPO_4_ · 4H_2_O concentration of 17.5 g L^−1^; Culture **D**: NZ-amine amount was increased by 5 fold, leading to 5 g L^−1^; Culture **E**: three times more trace elements were added, leading to 3 mL L^−1^; Culture **F**: three times more magnesium was added, leading to 3 mM of MgSO_4_ · 7H_2_O. The data are the average numbers of duplicates.

*E. coli* JM109 (pKSSE5.3) was cultivated in 1 L bioreactors containing modified E2 minimal medium. Various factors were tested for their influence on P4HB synthesis: carbon, nitrogen and amino acid source, trace elements and magnesium. As described previously [16], three phases (growth, accumulation and stagnation phase) were observed for cultures A (with standard medium containing modified E2 medium + 10 g L^−1^ xylose + 4 g L^−1^ Na-4HB + 1 g L^−1^ NZ-amines + 1 mL L^−1^ trace elements), B (two times more xylose), C (five times more nitrogen source NaNH_4_HPO_4_ · 4H_2_O), E (three times more trace elements), and F (three times more magnesium), whereas culture D (five times more NZ-amines) exhibited no accumulation phase (Figure 1). Culture D reached a maximal OD_600_ and a maximal P4HB content of about 8.6 and 3% (w w^−1^), respectively. Culture A with the standard medium led to highest maximal P4HB content of 65% (w w^−1^), while Cultures B, C, E and F reached a slightly lower maximal P4HB content of 52%, 52%, 59%, 45%, respectively.

These results showed that NZ-amines (amino acids) in excess blocked P4HB synthesis, whereas increased concentrations of carbon source, nitrogen source NaNH_4_HPO_4_ · 4H_2_O, trace elements or magnesium did not impact P4HB synthesis significantly. Normally, PHAs accumulate in the bacterial growth phase under nitrogen, phosphorous or oxygen limited conditions with an excess of carbon source [4,25]. It has been reported that recombinant *E. coli* does not require any nutrient limitation for synthesis of poly(3-hydroxybutyrate) (P3HB) and produces P3HB in a growth-associated manner even under nutrient-sufficient conditions [17]. In this study with a recombinant *E. coli* strain, neither nitrogen nor carbon source in excess led to a significant reduction of P4HB content, whereas excess of amino acids (NZ-amines) almost abolished P4HB synthesis (Figure 1). It seems that amino acid limitation caused a halt of cell growth and triggered P4HB accumulation.

Previously, we have tested a defined medium without addition of any amino acid for P4HB synthesis and found that the chemically defined medium resulted in hardly any P4HB synthesis [26]. Addition of a small amount of complex nitrogen sources such as NZ-amines promoted considerably P4HB accumulation [26]. Therefore, other means than omitting amino acid in the medium are needed to limit the intracellular amino acid concentration for promoting P4HB synthesis.

### Influence of acetate on P4HB synthesis

**Figure 2:**
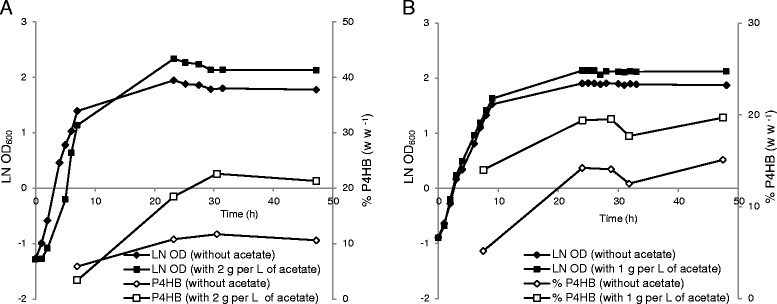
***E*** **.** ***coli*** **JM109 (pKSSE5.3) grown in modified E2 medium supplemented with or without acetate in shake flasks.** 10 g L^−1^ glycerol was used as the main carbon source. **A**: 2 g L^−1^ acetate was added to the culture; **B**: 1 g L^−1^ acetate was added to the culture. The data are the average numbers of duplicates.

To artificially obtain amino acid limitation, one possibility is to add weak organic acids to the culture medium. It has been reported that the growth inhibitory effect of acetic acid on *E. coli* is due to its influence on the amino acid (e.g. methionine) pool in the cells: the more acetic acid produced, the smaller the methionine pool becomes, leading to restriction of cell growth [27]. Recently we have reported that addition of propionic acid to the culture medium stimulates P4HB accumulation in recombinant *E. coli* grown on glycerol. This stimulating effect was significantly weakened by addition of exogenous methionine but not by cysteine, suggesting that propionic acid enhances P4HB synthesis at least partially by reducing the intracellular methionine pool [26]. Whether propionic acid also influences other amino acids pools is not investigated, thus not known. In this study, we further investigated whether the extracellular addition of acetic acid would enhance P4HB synthesis. Glycerol is a simple polyol compound and a side product from the biodiesel industries. *E. coli* grown on glycerol generates lower amounts of acetic acid than on xylose or glucose [15,28]. *E. coli* JM109 (pKSSE5.3) was grown in 1 L shake flasks containing modified E2 medium. A concentration of 10 g L^−1^ of glycerol was added with or without 2 g L^−1^ acetic acid at the beginning of the cultivation. With acetic acid a maximal content of 23% w w^−1^ P4HB was obtained, whereas without only 12% w w^−1^ was achieved (Figure 2A). To confirm that the observed enhanced P4HB content was not caused by a reduced growth rate due to the addition of acetic acid, 1 g L^−1^ instead of 2 g L^−1^ acetic acid was added at the beginning of the cultivation on glycerol. The cultures with or without 1 g L^−1^ acetic acid showed the same growth rate of 0.31 h^−1^ (Figure 2B); however, the culture with addition of acetic acid accumulated much more P4HB than the one without (Figure 2B). Thus, it can be speculated that the P4HB synthesis is stimulated by acetic acid addition through reduction of the intracellular amino acid pool rather than a reduction in specific growth rate, similar to the findings reported previously [26].

Previously, it has been reported that the molar fraction of 4HB in the P(3HB-*co*-4HB) biosynthesis by *R. eutropha* was increased significantly from 38 to 54 mol% by the addition of a small amount of acetic acid or propionate [29]. The authors suggested that acetate is able to increase acetyl-CoA pool, inhibit the ketolysis of 4-hydroxybutyryl-CoA to two molecules of acetyl-CoA, and consequently increase 4HB fraction. If this hypothesis is valid for *E. coli*, *E. coli* (pKSSE5.3) would be able to utilize 4HB as a sole carbon source for cell growth. However, *E. coli* JM109(pKSSE5.3) is not able to grow on medium containing 4HB as the sole carbon source and cannot use 4HB as a growth substrate even when combined with another growth C-source [16]. Furthermore, we have recently showed that propionic acid enhances P4HB synthesis by reducing the intracellular methionine pool [26]. Therefore, the hypothesis that addition of acetate stabilizes 4-hydroxybutyryl-CoA from ketolysis and consequently leads to a higher 4HB fraction in polymers is not valid here. The results obtained further confirmed the hypothesis reported in our previous work [16] that the pathways for cell growth and P4HB synthesis compete with each other. When the available nutrients and energy are used for cell growth, P4HB can hardly be synthesized. When the cell growth slows down/stops due to nutrient limitation (e.g. amino acids) other than carbon starvation, P4HB synthesis can be initiated. It has been reported that exogenous addition of acetic acid increases the acetyl-CoA synthetase (ACS) activity in order to reach the equilibrium between the concentration of acetate and acetyl-CoA [30]. An overflow of acetyl-CoA, which is the the donor of CoA to 4HB, increases the accumulation of P4HB.

### Influence of acetate addition on P4HB synthesis at different physiological growth stages

**Figure 3:**
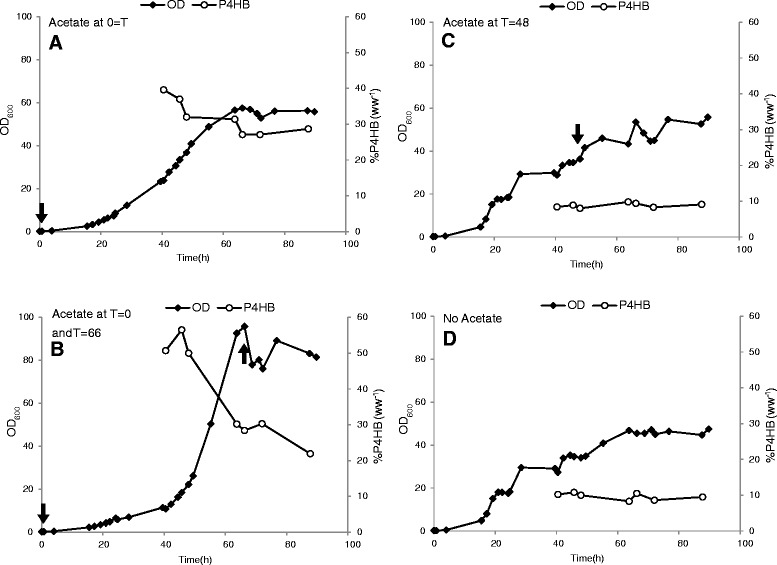
**Fed-batch strategy using acetic acid as stimulator for P4HB synthesis in** ***E*** **.** ***coli*** **JM109 (pKSSE5.3) grown on modified M9 medium supplemented with 20 g L** ^**−1**^ **glycerol, 6 g L** ^**−1**^ **Na-4HB, 0.5 g L** ^**−1**^ **NZ-amine, 0.015 g L** ^**−1**^ **thiamine and 100 mg L** ^**−1**^ **ampicillin.** Acetic acid was added at different physiological states. For all cultures pulse-feeding started at 40 h of cultivation: T = 40.5 h, addition of 12 g L^−1^ glycerol and 6 g L^−1^ Na-4HB; T = 45.75 h, addition of 20 g L^−1^ glycerol; T = 63.75 h, addition of 10 g L^−1^ glycerol and 3 g L^−1^ Na-4HB; T = 72.25 h, addition of 20 g L^−1^ glycerol and 6 g L^−1^ Na-4HB; T = 76.75 h, addition of 10 g L^−1^ glycerol. Culture **A**, addition of 2 g L^−1^ acetic acid at the beginning of the cultivation; Culture **B**, addition of 1 g L^−1^ acetic acid at beginning and at the end of growth phase (66 h), respectively; Culture **C**, addition of 2 g L^−1^ acetic acid after 48 h cultivation; Culture **D**, no addition of acetic acid to the culture. Arrows represent the addition of acetic acid. The data are the average numbers of duplicates.

The influence of acetic acid addition at different physiological growth stages was studied during high cell density cultivation. *E. coli* JM109 (pKSSE5.3) was grown on modified M9 medium. Glycerol and Na-4HB were pulsed when needed during cell growth, which was indicated by an increase of dissolved oxygen tension (pO_2_) signal. In culture A (Figure 3), 2 g L^−1^ of acetic acid was added at the beginning of the cultivation. The cells reached a maximal OD_600_ of 57.5 with a P4HB content of 31% w w^−1^ at 64 h of cultivation. In culture B, 1 g L^−1^ acetic acid was added twice, first at the beginning and again at the end of the growth phase (66 h). The cells reached a maximal OD_600_ of 92.6 with a P4HB content of 30% w w^−1^ at 63 h. In culture C, 2 g L^−1^ acetic acid was added at 48 h of cultivation. The cells reached a maximal OD_600_ of 53.5 at 66.25 h with a P4HB content of 9% w w^−1^. The culture without any addition of acetic acid (culture D) reached a maximal OD_600_ of 45.5 with a P4HB content of 10% w w ^−1^ (Figure 3). These results demonstrated that the addition of acetic acid at the beginning of the cultivation enhances P4HB accumulation dramatically, leading to a three-fold higher P4HB content than without acetic acid. The addition of acetic acid at the end of the exponential growth phase did not result in any improvement of P4HB production compared to the culture without any acetic acid.

The reason why addition of acetic acid at the end of growth phase did not promote P4HB synthesis could be that cell metabolism at the stationary phase is not active enough to convert acetic acid to acetyl-CoA. When acetic acid is added at the beginning of the growth phase, it can be converted to acetyl-CoA which can be further channeled to cell growth and maintenance (during the growth phase) or P4HB synthesis (during the accumulation phase).

### Influence of the feeding mode on P4HB product during fed-batch culture

Based on the above results, different nutrient feeding strategies were compared for P4HB production in recombinant *E. coli* JM109 (pKSSE5.3) using glycerol as the carbon substrate and acetic acid as the stimulator.

#### Pulse-feeding

**Figure 4:**
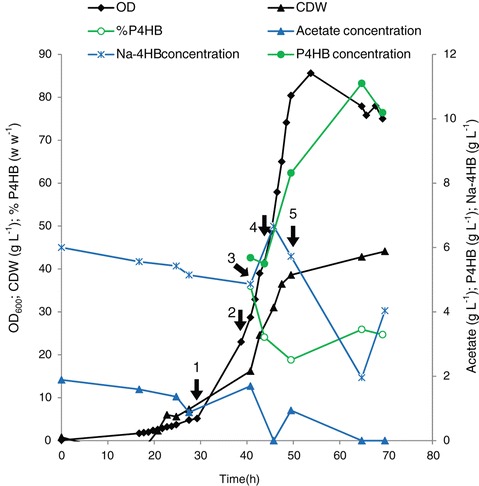
**Time course of cell dry weight (CDW), P4HB content and P4HB concentration during a pulse feeding fed-batch culture of** ***E*** **.** ***coli*** **JM109 (pKSSE5.3).** Glycerol and 4-hydroxybutyric acid were used as carbon source and precursor, respectively. Cultivation was conducted at 32°C in a 1 L bioreactor with an initial volume of 600 mL of modified M9 medium plus 20 g L^−1^ glycerol, 6 g L^−1^ Na-4HB, 2 g L^−1^ acetic acid, 0.5 g L^−1^ NZ-amines, 0.015 g L^−1^ thiamine and 100 mg L^−1^ ampicillin. Feeding solution was added to the bioreactor when glycerol in the medium was close to depletion, indicated by pO_2_ signal. Arrow #1, feeding of 12.5 g L^−1^ glycerol + 2.5 g L^−1^ Na-4HB + 1.1 g L^−1^ acetate at 30 h; Arrow #2, feeding of 25.0 g L^−1^ glycerol + 2.1 g L^−1^ acetate at 39 h; Arrow #3, feeding of 2.5 g L^−1^ Na-4HB at 41 h; Arrow #4, feeding of 25.0 g L^−1^ glycerol + 2.1 g L^−1^ acetate + 2.5 g L^−1^ Na-4HB at 44 h; Arrow #5, feeding of 25.0 g L^−1^ glycerol + 2.1 g L^−1^ acetate + 6 g L^−1^ Na-4HB at 49.5 h. The data are the average numbers of duplicates.

The batch culture was performed using modified M9 medium. Glycerol, Na-4HB and acetic acid were added when the carbon source glycerol was limited. Glycerol limitation was monitored by pO2 signal as described in Materials and Methods. This culture grew with an initial specific growth rate of 0.11 h^−1^ (Figure 4). The concentration of Na-4HB was never limiting and did not exceed 7 g L^−1^. The maximal Na-4HB consumption rate was 0.43 g L^−1^ h^−1^, leading to a maximal specific consumption rate of 0.05 g g^−1^ h^−1^. The initially fed acetic acid was not totally consumed when the first pulse of acetic acid was added to the culture broth after 30 h of cultivation. No visible impact on the cell growth was observed after this addition. After 39 h of cultivation, acetic acid was added once more, which was consumed quickly with a specific consumption rate of 0.032 g g^−1^ h^−1^. The P4HB content decreased after 40 h of cultivation along with the cell growth. However, the P4HB concentration continuously increased, for example, during 9 h of cultivation from 40 h to 49 h 2.63 g L^−1^ P4HB was accumulated, leading to a P4HB accumulation rate of 0.25 g L^−1^ h^−1^. The OD_600_ increased continuously to 86 within 53 h, afterwards decreased to 75 during prolonged cultivation from 53 h to 69 h (Figure 4).

#### Linear-feeding

The cells were grown in modified M9 medium. A feeding solution containing 200 g L^−1^ acetic acid and 200 g L^−1^ glycerol was used for the first 65 h of cultivation and then exchanged with a feeding solution of 100 g L^−1^ acetic acid and 400 g L^−1^ glycerol for the next 69 h. Different feed rates were compared: 0.5, 1, 2 and 3 mL h^−1^. It was found that the best feed rate for P4HB synthesis was between 1 and 2 mL h^−1^ under the conditions used in this study. Below or above this range P4HB content decreased. Thus, the feed rates of 1 and 2 mL h^−1^ were studied in more details.

**Figure 5:**
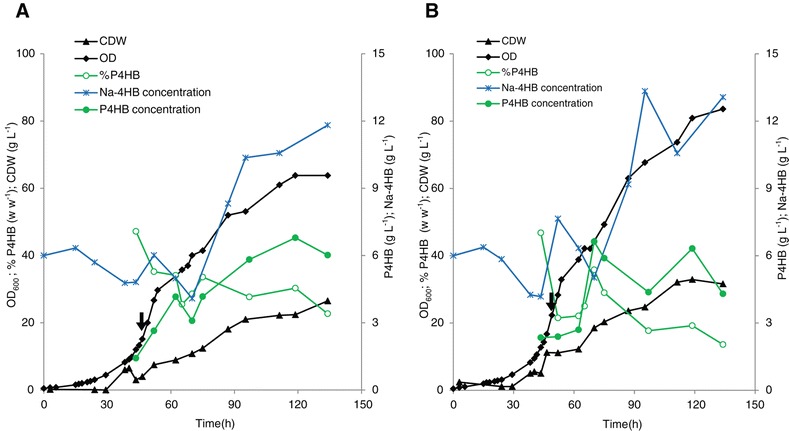
**High cell density cultivation with linear feeding mode of** ***E*** **.** ***coli*** **JM109 (pKSSE5.3).** The feeding solution contained 200 g L^−1^ acetic acid and 200 g L^−1^ glycerol for 65 h and then 100 g L^−1^ acetic acid and 200 g L^−1^ glycerol. Panel **A**: feeding rate of 1 mL h^−1^; **B**: feeding rate of 2 mL h^−1^. Arrows represent the start of feeding. The data are the average numbers of duplicates.

Figure 5 shows that with the feeding rate of 1 mL h^−1^ (Culture A) the cells reached a maximal OD_600_ value of 63.8, a CDW of 22.4 g L^−1^ and a P4HB content of 30% w w^−1^ after 119 h, leading to a product concentration of about 7 g L^−1^ P4HB. In Culture B (feeding rate of 2 mL h^−1^), the cells reached a maximal OD_600_ value, a CDW and a P4HB content of 80.9, 32.9 g L^−1^, and 19% w w^−1^ after 119 h, respectively, leading to a yield of about 6 g L^−1^ P4HB. For both cultures, the Na-4HB precursor was not limiting, however, the P4HB content decreased dramatically after 40 h of cell growth. It seems that most of the cells generated during the late stage had difficulty to accumulate P4HB; leading to a dilution of the P4HB content caused by cell divisions even if the overall P4HB concentrations were increased. Previously, Song and co-workers have reported similar phenomenon that almost no P4HB accumulated in newly-produced cells in the late stage during a fed-batch experiment [31]. No explanation could be given. It cannot be caused by the plasmid instability because at the end of the cultivations cells were taken and plated on LB agar with or without ampicillin and were found to maintain at least 90% of the plasmid (data not shown).

#### Exponential-feeding

**Figure 6:**
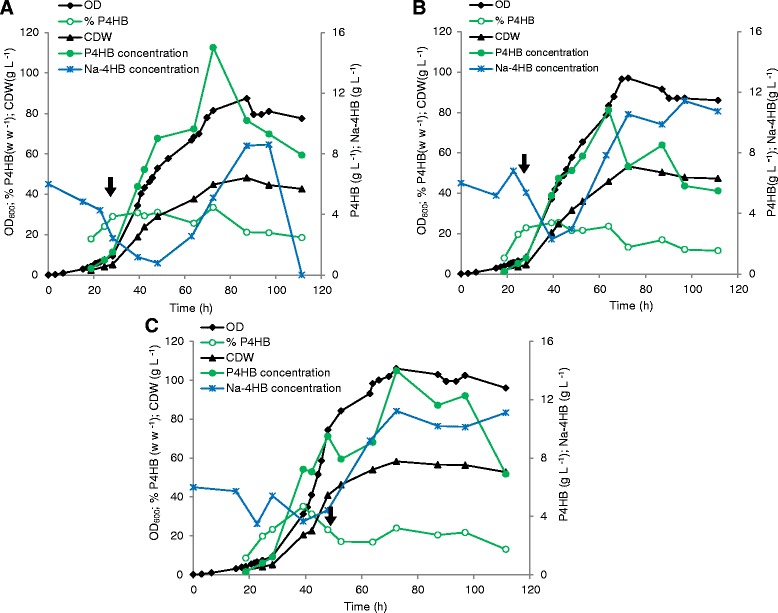
**High cell density cultivation with exponential feeding mode.** The feeding solution contained 40 g L^−1^ Na-4HB, 300 g L^−1^ glycerol, and 20 g L^−1^ acetic acid. Controlled feeding rate was set for the cultures **A**, **B** and **C** at 0.02, 0.04 and 0.08 h^−1^, respectively. Arrows represent the start of feeding. The data are the average numbers of duplicates.

The batch culture was conducted using modified M9 medium. Three different dilution rates of 0.02 h^−1^, 0.04 h^−1^ and 0.08 h^−1^ were tested in cultures A, B and C, respectively. The feeding solution used for all three cultures was composed of 40 g L^−1^ Na-4HB, 300 g L^−1^ glycerol and 20 g L^−1^ acetic acid. The culture A reached a higher maximal OD_600_ of about 80 after 72.5 h of cultivation (Figure 6). Cultures B and C showed a similar maximal OD_600_ of about 100 after 72.5 h of cultivation (Figure 6). No Na-4HB precursor limitation was observed for any of the three cultivations. The exponential growth stopped at about 53 h, even though glycerol, nitrogen, acetic acid and Na-4HB were found to be still available in the medium based on the measurement described in the Materials and Methods (data not shown). A maximal P4HB content of 34% w w^−1^ and a P4HB concentration of 15 g L^−1^ were obtained for culture A after 72.5 h of cultivation. These results demonstrate that concomitant addition of acetic acid, glycerol and Na-4HB precursor in *E. coli* JM109 (pKSSE5.3) can lead to a high productivity by using a slow exponential feeding.

In summary, with the pulse feed strategy an addition of acetic acid at the beginning of the cultivation led to a multi-fold increase in P4HB yield from 9% to 31% (w w^−1^) (Figure 3). A yield of 11.1 g L^−1^ and a P4HB productivity of 0.173 g L^−1^ h^−1^ within 64 h could be achieved. With a linear feeding mode a lower yield was obtained than with pulse feeding (Table 1). The best feeding rate was 1 mL h^−1^, leading to a P4HB yield of 6.8 g L^−1^ and a productivity of 0.058 g L^−1^ h^−1^ over 118 h. Exponential feeding led to the maximal yield of 15 g L^−1^ P4HB in about 72.5 h with a feeding rate of 0.02 h^−1^, resulting in a productivity of 0.207 g L^−1^ h^−1^ (Table 1). Previously, it has been reported that the production of P4HB homopolymer using glucose as growth substrate and Na-4HB as precursor can reach a final yield of 4.0 g L^−1^ and a P4HB productivity of 0.065 g L^−1^ h^−1^ in 62 h *via* a pulse feeding strategy [31]. A recent publication showed that an *E. coli* recombinant JM109SG carrying two plasmids could utilize solely glucose for P4HB production [32], and a P4HB yield of 7.8 g L^−1^ and a productivity of 0.150 g L^−1^ h^−1^ were obtained by using LB medium containing yeast extract in a pulse feeding fed-batch culture [32]. The productivity of 0.207 g L^−1^ h^−1^ obtained in this study is the highest reported so far.

**Table 1:** **Summary of different feeding modes and their effect on P4HB production during fed**-**batch cultivations**

**Growth substrate/stimulator/precursor/media**	**Feeding strategy**	**Culture time (h)**	**OD**_**600max**_	**CDW (g L** ^**−1**^ **)**	**P4HB content% (w w** ^**−1**^ **)**	**Volumetric yield P4HB (g L** ^**−1**^ **)**	**Productivity (g L** ^**−1**^ **h** ^**−1**^ **)**	**References**
Glycerol/acetate/Na-4HB/modified M9 medium	Pulse feed	64	77.9	42.8	26	11.5	0.180	This study
	Linear feed	118	63.8	33.8	30	6.8	0.058	
	Exponential feed	73	81.5	43.2	33	15.0	0.207	
Glucose/-/4HB/M9 medium	Pulse feed	62	24.5	13.0	31	4.0	0.065	[31]
Glucose/-/yeast extract/LB medium	Pulse feed	52	21.7	11.5	68	7.8	0.150	[32]

In this study, we also demonstrated that even though the cost of Na-4HB is relative high, it can be significantly reduced by using gamma-butyrolactone as a low-cost precursor for chemical synthesis of Na-4HB (see Methods section). Furthermore, Na-4HB was not used as the growth substrate but the precursor for P4HB, thus only low amount was needed, e.g. a total of about 19 g L^−1^ Na-4HB was added to produce 11 g L^−1^ P4HB in the case of pulse feeding fed-batch culture (Figure 4).

To further improve the productivity and reduce the cost of P4HB one of the imperative tasks is to achieve P4HB accumulation in newly-produced cells in the late stage during a fed-batch experiment, thus avoiding the dilution of P4HB content.

## Conclusions

In this study, we demonstrated that the NZ-amines (amino acids source) in excess abolished P4HB accumulation, suggesting that limitation in certain amino acid pools promotes P4HB synthesis. This was validated by providing exogenous acetic acid to the cells, which most likely resulted in the reduction of the intracellular amino acid pool. Furthermore, the enhanced P4HB yield was achieved by both the effective growth of *E. coli* JM109 (pKSSE5.3) on glycerol and the stimulated P4HB synthesis *via* exogenous addition of acetic acid. We have developed fermentation strategies for P4HB production by using glycerol, leading to a productivity of 0.207 g L^−1^ h^−1^ P4HB, which is the highest yield for P4HB production reported so far. This high P4HB productivity will decrease the total production cost, allowing further development of P4HB applications.

## Methods

### Bacterial strain and plasmid

*Escherichia coli* JM109 [33] carrying plasmid pKSSE5.3 was used throughout the whole study. pKSSE5.3 harbors the PHA synthase gene (*phaC*) from *Ralstonia eutropha* and a 4-hydroxybutyric acid-coenzyme A transferase gene (*orfZ*) from *Clostridium kluyveri* [34], and enables *E. coli* strains to produce P4HB when 4HB is supplied in the culture medium. The expression of *phaC* and *orfZ* on pKSSE5.3 is driven by their native promoter(s) [34].

### Chemicals

All chemicals were purchased from Sigma-Aldrich (Buchs, Switzerland).

### Synthesis of sodium 4-hydroxybutyrate (Na-4HB)

Na-4HB was synthesized by hydrolysis of the corresponding lactone. The synthesis was performed as describe previously [16,35,36]. In detail, a 4 M NaOH solution was prepared and mixed slowly with 4 M of beta-butyrolactone on ice. The reaction mixture was cooled down to room temperature and analyzed by HPLC/MS [16,35]. An almost 100% conversion of beta-butyrolactone to Na-4HB was achieved.

### Media and cultivation conditions

#### Shake flasks experiments

Growth studies were performed in 1 L shake flasks containing 200 mL of modified E2 medium and 10 g L^−1^ of carbon source glycerol. One g L^−1^ of NZ-amines, 100 μg mL^−1^ ampicillin and 4 g L^−1^ of Na-4HB were added at the beginning of the cultivation. NZ-amines are casein enzymatic hydrolysates with a total amino acid content of approximately 0.89 g g^−1^. Cultures were incubated at 32°C and 150 rpm based on our previous study [16]. Modified E2 medium was composed of the following components: NaNH_4_HPO_4_ · 4H_2_O 3.5 g L^−1^, KH_2_PO_4_ 3.7 g L^−1^ and K_2_HPO_4_ 7.5 g L^−1^ dissolved in distilled water. One mL L^−1^ of 1 M MgSO_4_ · 7H_2_O and 1 mL L^−1^ of trace elements (TE) dissolved in 1 M HCl were added. TE contains FeSO_4_ · 7H_2_O 2.78 g L^−1^, CaCl_2_ · 2H_2_O 1.47 g L^−1^, MnCl_2_ · 4H_2_O 1.98 g L^−1^, CoCl_2_ · 6H_2_O 2.38 g L^−1^, CuCl_2_ · 2H_2_O 0.17 g L^−1^, and ZnSO_4_ · 7H_2_O 0.29 g L^−1^. LB was used as the preculture medium to inoculate the main culture to an initial OD_600_ between 0.2 and 0.3.

### Bioreactor experiments

#### Experiments of identification of influencing factors in batch culture

*E. coli* JM109 (pKSSE5.3) cells were grown at 32°C in 1 L bioreactors (Infors AG, Bottmingen, Switzerland) containing modified E2 medium supplemented with 10 g L^−1^ xylose, 4 g L^−1^ Na-4HB, 1 g L^−1^ NZ-amines and 0.015 g L^−1^ thiamine. Preculture medium had the same composition as the one for the main culture. The initial OD_600_ value in bioreactors was always between 0.1 and 0.3 units. Temperature was controlled at 32°C and pH was maintained at 7.0 by automated addition of 25% NaOH or 2 M H_2_SO_4_. The dissolved oxygen tension was monitored continuously with an oxygen probe and maintained at 30% of oxygen saturation.

#### High cell density culture experiments

In order to improve the productivity, high cell density cultivations were performed using *E. coli* JM109 (pKSSE5.3). Modified M9 medium instead of modified E2 medium was used in these studies because modified M9 medium was reported to be suitable for high cell density culture of *E. coli* JM109 [37]. Modified M9 medium contained 4 g L^−1^ (NH_4_)_2_HPO_4_, 13.3 g L^−1^ KH_2_PO_4_, 1 g L^−1^ (NH_4_)_2_SO_4_, 20 g L^−1^ glycerol, 6 g L^−1^ Na-4HB, and 0.5 g L^−1^ NZ-amines. After autoclaving the medium, 10 mL L^−1^ of trace elements composed of 2.5 g L^−1^ CaCl_2_, 0.075 g L^−1^ CuCl_2_ · 4H_2_O, 3.525 g L^−1^ FeCl_3_ · 4H_2_O, 0.65 g L^−1^ Zn(CH_3_COO)_2_, 0.75 g L^−1^ MnCl_2_ · 4H_2_O, 0.125 g L^−1^ CoCl_2_ · 6H_2_O, 0.15 g L^−1^ H_3_BO_3_, 0.125 g L^−1^ NaMoO_4_ · 2H_2_O and 0.625 g L^−1^ Na_2_EDTA were added to the medium. In addition, 5 mL L^−1^ of 1 M MgSO_4_ · 7H_2_O, 0.015 g L^−1^ thiamine, and 100 mg L^−1^ ampicillin were filter sterilized separately and added to the bioreactors before inoculation. Preculture medium had the same composition as the one for the main culture. The initial OD_600_ value in bioreactors was always between 0.1 and 0.3 units. Temperature was controlled at 32°C and pH was maintained at 7.0 by automated addition of 7.7 M NH_4_OH or 2 M H_2_SO_4_. The dissolved oxygen tension was monitored continuously with an oxygen probe and maintained at 30% of oxygen saturation.

For exponential feedings, the substrate feeding rate (*F*) for controlling the specific growth rate (μ) was determined as follows with neglecting the carbon substrate consumption for cell energy maintenance. To get a time-dependent exponential feed, it is necessary to achieve a constant μ that is lower than μ_max_. We started from the mass balance on the limiting substrate, which in our case is the growth carbon substrate (glycerol). The consumption of growth limiting substrate concentration according to the time can be expressed by:

1 $$ \frac{dS}{dt}=\frac{F}{V}\ \left({s}_0-s\right)-{q}_s\ x $$

where *s*_0_ is the limiting substrate concentration (g L^−1^) in feeding medium and *s* is the actual growth limiting substrate concentration (g L^−1^) in culture broth, *Y*_*X*/*S*_ is the growth yield (g g^−1^) for the limiting substrate and *q*_*s*_ is the specific substrate consumption rate (g g^−1^ h^−1^).

Because the cell density in the fed-batch is very high and *s*_0_ therefore consumed rapidly, it can be stated that *s* < < *s*_0_ and $\frac{ds}{dt}\approx 0$. Consequently, equation 1 can be modified to:

2 $$ F=\frac{q_s\ (xV)}{s_0} $$

The biomass concentration (*x*) and the volume of the culture (*V*) increased with time, leading to:

3 $$ {(xV)}_t = \left({x}_0\ {V}_0\right){e}^{\upmu t} $$

Where *x*_0_ and *V*_o_ are starting biomass concentration and the initial volume of culture, respectively. Hence, the flow rate which enables recombinant *E. coli* to grow at a constant μ is obtained in equation (4) by combining the equations (2) and (3).

4 $$ F(t)=\frac{q_s}{s_0}\ \left({x}_0{V}_0\right){e}^{\upmu t} $$

This is equivalent to:

5 $$ F(t) = {F}_0{e}^{\upmu t} $$

This means one can formulate the starting flow condition *F*_o_ at *t* = 0 h as follows:

6 $$ {F}_0=\frac{\upmu}{s_0{Y}_{X/S}}\ {x}_0 $$

7 $$ {F}_0=\upmu\ V $$

The exponential feeding technique allows controlling the overflow metabolism of recombinant *E. coli* in a fed-batch process. This technique makes it possible to grow the culture at a constant specific growth rate and consequently the yield coefficient *Y*_*X*/*S*_ remains constant.

### Test of plasmid stability

Cells at the end of cultivation were collected and a serial dilution of the cell suspension was prepared. The suspensions were plated on the LB agar plate with or without ampicillin. The plates were incubated overnight at 37°C and the colony numbers on plates with and without ampicillin were counted and compared.

### Analytical methods

#### Cell concentration

Growth of bacterial cells was followed by measuring optical density at 600 nm (OD_600_) using a UV-visible spectrophotometer (Genesys 6, ThermoSpectronic, Switzerland).

Cell dry weight (CDW) was determined using 2 mL pre-weighted Eppendorf tubes. Two mL culture broth were added into the tube and centrifuged at 10’000 g for 2 min. The cell pellet was washed once with water. Cells were spun down again and the cell pellet was dried overnight at 100°C, cooled down to room temperature in a desiccator and weighed. The weight difference was used to determine the quantity of biomass per culture volume.

#### PHA content

To determine the PHA content and composition, the culture was centrifuged (8’500 g, 4°C, 15 min) and the cell pellet was washed once with water and lyophilized for 48 hours. Biomass in the range of 20–50 mg was added to Pyrex vials. Then, 2 ml of 15% v v^−1^ H_2_SO_4_ in methanol was added and mixed. Furthermore, 2 ml of methylene chloride containing benzoic acid (0.1 g L^−1^) as internal standard were added. The suspension was boiled at 100°C for 2.5 h in an oven. The samples were cooled on ice, and 1 ml of distilled water was added in order to extract the cell debris into the aqueous phase. The solution was mixed by vortexing for 1 min. The complete (upper) water phase was discarded, including droplets hanging on the tube wall. The remaining methylene chloride phase was dried and neutralized by adding Na_2_SO_4_ and Na_2_CO_3_ powder, and 200 μl of the organic phase were filtered using a solvent resistant filter (PTFE, 0.45 μm) and transferred to a GC sample vial. Samples were analyzed using gas chromatography (GC) (A200s, Trace GC 2000 series, Fisons Instruments, Rodano, Italy) equipped with a polar fused silica capillary column (Supelcowax-10: length 30 m; inside diameter 0.31 mm; film thickness 0.5 μm; Supelco, Sigma-Aldrich, Buchs, Switzerland) [38]. The methylation of P4HB resulted in 3 distinct peaks representing the methylester of 4HB, γ-butyrolactone and the methyl ether of 4HB, respectively, which were also obtained if only Na-4HB was subjected to methanolysis. These three peaks were also observed by others when analyzing P4HB homopolymers [16,34,39].

### Evaluation of glycerol limitation

The dissolved oxygen tension (pO_2_) was used as an indicator for glycerol consumption during fed-batch cultures [40]. This is based on the fact that whenever the substrate in the medium is about to run out and thus becomes a limiting factor, the pO_2_ increases rapidly. When the carbon substrate is added to the culture, pO_2_ decreases to its former level.

### Measurement of nitrogen

NH_4_^+^-nitrogen content was measured using an ammonium test kit following the manufacturer instruction (Merck KGaA, 64271 Darmstadt, Germany). The detection range was from 0.01 to 3.0 NH4^+^-N mg L^−1^, above which dilution with distilled water was needed.

### Acetate and Na-4HB measurements

Acetate and Na-4HB were measured by HPLC/MS (Agilent 1000 Series, Santa Clara, United States for the HPLC unit, and Bruker Daltonics esquire HCT, Bremen, Germany for the MS unit). Supernatant resulting from culture centrifugation at 10’000 g for 2 min was diluted to 0.01 to 0.1 mM with distilled water and loaded on a reversed phase C18 column (Gemini C18 5 micron, 250 mm × 4.60 mm, Phenomenex, U.K.). A gradient of diluted formic acid (0.1% v v^−1^ in water) to 100% acetonitrile mixed with 0.1% v v ^−1^ formic acid was applied as the mobile phase. The flow rate was 0.8 mL min^−1^ and the gradient was completed after 25 minutes. The peaks were detected by electrospray ionization (ESI) in negative mode [35]. The standard curves for acetate and Na-4HB were recorded in the range of 0.01 to 1 g L^−1^ and 0.01 to 0.2 g L^−1^, respectively.

### Reproducibility

In this study, for each batch culture at least two independent experiments were performed, for each fed-batch culture at least three independent experiments were performed. The absolute values of cell density and P4HB content obtained from the independent experiments varied, which is not surprising for biological systems. This could be caused by slight differences in inoculum, cultivation conditions, sampling, and etc. However, the cell growth and P4HB synthesis exhibited same patterns in the same set of independent experiments. In this report the results obtained from one independent experiment were presented. Each individual sample was measured in duplicates. The data presented here are the average numbers.
